# Dedicated Bifurcation Stents vs. Regular Drug-Eluting Stents in Coronary Bifurcation Treatment: A Systematic Review and Meta-Analysis of 1-Year and 4-Year Outcomes, Including Left Main and Non-Left Main Subgroup Comparisons

**DOI:** 10.3390/biomedicines13112763

**Published:** 2025-11-12

**Authors:** Jacek Bil, Adam Kern, Aneta I. Gziut-Rudkowska, Jarosław Zalewski, Krystian Bojko, Robert J. Gil

**Affiliations:** 1 Department of Cardiology, National Medical Institute of the Ministry of Interior and Administration, 02-507 Warsaw, Poland; jacek.bil@pimmswia.gov.pl (J.B.);; 2 Department of Cardiology and Internal Medicine, School of Medicine, Collegium Medicum, University of Warmia and Mazury, 10-719 Olsztyn, Poland; 3 Department of Coronary Artery Disease and Heart Failure, Institute of Cardiology, Jagiellonian University Medical College, 31-008 Kraków, Poland

**Keywords:** sirolimus-eluting stent, coronary artery disease, coronary bifurcation, dedicated bifurcation stent

## Abstract

**Background**: Dedicated bifurcation stents (DBS) were developed to overcome the limitations of conventional drug-eluting stents (DES) in percutaneous coronary intervention (PCI) for bifurcation lesions, but their clinical benefit remains uncertain. **Methods**: We conducted a systematic review and meta-analysis of randomized trials comparing DBS with contemporary DES in bifurcation PCI. Primary outcomes included all-cause death, myocardial infarction (MI), and target lesion revascularization (TLR) at 1 and 4 years. Subgroup analyses were performed for left main (LM) and non-LM bifurcations. **Results**: Ten trials involving approximately 2500 patients were analyzed. At 1 year, DBS and DES demonstrated similar rates of all-cause death (RR 1.12, 95% CI 0.81–1.55), MI (RR 0.80, 95% CI 0.38–1.69), and TLR (RR 1.23, 95% CI 0.79–1.90). At 4 years, results remained consistent: all-cause death (RR 1.10, 95% CI 0.75–1.60), MI (RR 0.66, 95% CI 0.29–1.49), and TLR (RR 1.29, 95% CI 0.86–1.94). No significant differences were observed between LM and non-LM subgroups, and no excess in late stent thrombosis was detected. **Conclusions**: DBS are safe and provide outcomes comparable to DES in bifurcation PCI. Their use may be reasonable in selected anatomies, but larger trials are needed to define their clinical advantage.

## 1. Introduction

Coronary bifurcation lesions (CBLs) represent one of the most complex challenges in interventional cardiology, accounting for approximately 15–20% of all percutaneous coronary interventions (PCIs). These lesions are characterized by their unique anatomy, which involves a division of the coronary artery into two branches, often complicating procedural planning and execution. Their prevalence and complexity necessitate specialized approaches to achieve optimal clinical outcomes while minimizing procedural risks [1,2].

The Provisional T-Stenting (PTS) technique, as recommended by the European Bifurcation Club, remains the preferred strategy for most bifurcation lesions due to its simplicity and comparable outcomes to more complex techniques [3]. However, two-stent techniques may be necessary for more complex or true bifurcation lesions—especially those involving the left main coronary artery. These techniques, such as Culotte or DK-Crush, are technically demanding and associated with higher procedural and fluoroscopy times, as well as greater risks of complications [4,5]. Complications such as SB loss, stent malposition, deformation, or fracture remain significant procedural challenges. In contrast, double-stent strategies, which involve the systematic implantation of stents in both the main vessel and SB, result in overlapping stent layers. This overlap increases the likelihood of neocarina formation and associated complications, such as periprocedural myocardial infarction (MI), stent thrombosis (ST), and the need for reintervention, highlighting the delicate balance between efficacy and safety in bifurcation PCI [6]. In this context, dedicated bifurcation stents were developed to simplify procedures, improve outcomes, and provide a specialized solution for these challenging lesions.

Dedicated bifurcation stents were initially introduced to overcome limitations associated with conventional drug-eluting stents (DES) in bifurcation PCI, such as suboptimal side-branch access and increased restenosis risk [7]. While earlier-generation dedicated bifurcation stents showed promise, they were often limited by complex delivery systems, challenging deployment techniques, and suboptimal clinical outcomes in large-scale studies [8]. However, recent iterations of dedicated bifurcation stents have demonstrated improved design and deliverability, translating into potential efficacy gains, reigniting interest in their role within bifurcation PCI. Early studies suggest that DBSs may simplify bifurcation lesion management and improve outcomes in select patients [9]. However, most investigations into DBS implantation are limited by small sample sizes and are underpowered to detect meaningful differences in key clinical outcomes, such as long-term patency and adverse events. Moreover, many studies are inherently biased, making it difficult to draw definitive conclusions about their broad applicability.

In detail, DBS technology has undergone substantial evolution, aiming to better conform to the complex geometry and hemodynamics of bifurcation lesions. The early BiOSS Expert platform introduced the concept of a stepped balloon and dual-diameter design to accommodate proximal–distal vessel tapering, improving main vessel (MV) and SB access. Its successor, the BiOSS LIM C, refined this concept with a thin-strut cobalt-chromium structure and a sirolimus-eluting coating, offering enhanced deliverability and radial strength [10]. Similarly, the Axxess self-expanding stent was engineered to provide conical scaffolding at the carina level, maintaining optimal flow distribution and minimizing vessel straightening. More recently, the Biomime Branch system has integrated an open-cell side-branch design that allows complete ostial coverage and easy SB rewiring while preserving laminar flow patterns [11]. These technological refinements reflect the continuous effort to align DBS architecture with the unique anatomical and physiological requirements of bifurcation PCI, potentially mitigating malapposition, neocarina formation, and flow disturbance.

Given the evolving role of dedicated bifurcation stents and the persistent challenge of treating bifurcation lesions, this systematic review and meta-analysis aims to compare their outcomes to those of conventional DES in treating coronary bifurcation lesions. By synthesizing data on 1-year and 4-year outcomes, including subgroup analyses for left main (LM) and non-left main bifurcations (non-LM), this study seeks to provide clarity on the efficacy and safety of dedicated bifurcation stents in contemporary clinical practice.

## 2. Materials and Methods

### 2.1. Data Sources and Searches

This systematic review was registered in the PROSPERO network (registration number: CRD42025632258). The systematic review protocol and reporting adhered to the Preferred Reporting Items for Systematic Reviews and Meta-Analyses (PRISMA 2020) guidelines [12]. Ethical approval was not required as this study involved the secondary collection and analysis of previously published data.

A systematic search was conducted in PubMed, Embase, Cochrane Library, Web of Science, and Google Scholar, covering the period from their inception to December 2024. The primary search terms included “dedicated bifurcation stent”, “coronary”, and “bifurcation”. Additionally, a manual search was performed to verify and identify relevant references within the selected articles. The search strategy used for PubMed is presented in Table 1. Two reviewers independently screened titles and abstracts for relevance. Full texts of potentially eligible studies were reviewed against the inclusion criteria. Disagreements were resolved by discussion or a third reviewer. Conference abstracts were excluded due to insufficient outcome data. The study selection process was documented using a PRISMA flow diagram.

### 2.2. Eligibility Criteria

We included randomized controlled trials (RCTs) or observational comparative studies enrolling adult patients (≥18 years) undergoing PCI for coronary bifurcation lesions, treated with either dedicated bifurcation stents or regular DES. Eligible studies compared dedicated bifurcation stents (DBS) with regular, non-dedicated drug-eluting stents (DES), regardless of whether the latter involved a provisional or planned two-stent technique (e.g., culotte, DK-crush, modified T-stenting). Eligible studies reported 1-year and/or 4-year outcomes and included patients with LM and/or non-LM bifurcation lesions. Exclusion criteria were: non-comparative case series, registry summaries without a control group, pediatric populations, congenital heart disease, non-bifurcation lesions, use of bare-metal or non-specified stent types, lack of subgroup data for LM/non-LM bifurcations, or insufficient outcome data.

### 2.3. Data Extraction and Quality Appraisal

Data extraction was conducted independently by two reviewers using a standardized form. Extracted data included study design, country, sample size, stent type, procedural characteristics, patient demographics, lesion location (LM vs. non-LM), follow-up duration, and clinical outcomes at 1 and 4 years. Outcomes extracted included:

- All-cause mortality
- Target lesion revascularization (TLR),
- Myocardial infarction,

The exact definitions of the study endpoints per study are presented in the Supplementary Table S1.

When necessary, study authors were contacted to clarify or complete data. Risk of bias was assessed independently by two reviewers. The Cochrane Risk of Bias 2 (RoB 2) tool was used. Assessments included aspects of randomization, blinding, attrition, outcome measurement, and selective reporting. Discrepancies were resolved through discussion.

### 2.4. Data Synthesis and Statistical Analysis

We performed this meta-analysis in accordance with the PRISMA 2020 guidelines. The primary effect measure was the risk ratio (RR) with the corresponding 95% confidence interval (CI), comparing DBS vs. DES. Meta-analyses were conducted using a random-effects model (DerSimonian–Laird method) to account for potential between-study variability, even if statistical heterogeneity was low. For comparison, a fixed-effect model was also explored in sensitivity analyses.

Statistical heterogeneity was quantified using the I^2^ statistic (with values of 25%, 50%, and 75% interpreted as low, moderate, and high heterogeneity, respectively) and Cochran’s Q test (*p* < 0.10 considered statistically significant). We also calculated τ^2^ (tau-squared) as an estimate of between-study variance. The review protocol pre-specified the conduct of sensitivity analyses using a fixed-effect model to assess the robustness of pooled results.

Potential publication bias was examined using a funnel plot and visually assessed for asymmetry. Due to the limited number of studies, formal tests for small-study effects (e.g., Egger’s regression) were not performed.

In addition, we performed a trial sequential analysis (TSA) to evaluate the robustness of the evidence and to determine whether the accrued sample size had reached the required information size. TSA was conducted with a two-sided α = 0.05, 80% power, and a relative risk reduction of 20%, applying O’Brien–Fleming monitoring boundaries to control for type I error due to repeated significance testing.

All analyses were conducted using RevMan (v5.4.1, Cochrane Collaboration, London, UK) for the meta-analysis and TSA software (version 0.9.5.10 Beta (Copenhagen Trial Unit, Copenhagen, Denmark)) for trial sequential analysis. A two-sided *p*-value < 0.05 was considered statistically significant, except where otherwise specified.

## 3. Results

### 3.1. Study Selection

**Figure 1:**
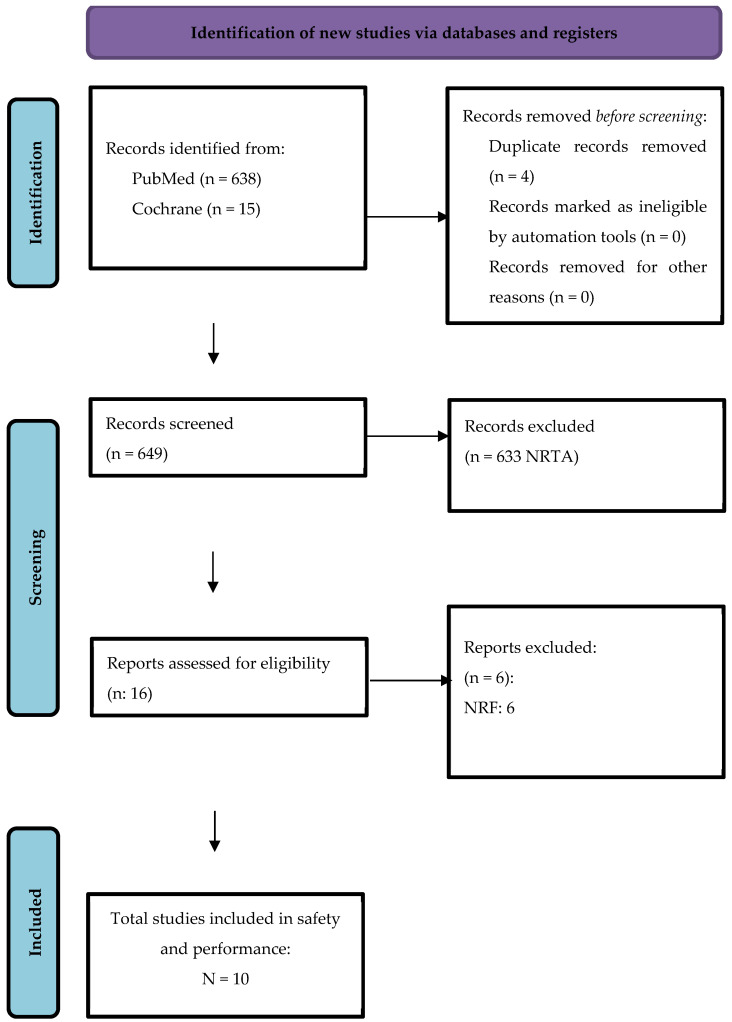
PRISMA flow chart. NRTA—not related text or abstract; NRF—not related fulltext.

**Figure 2:**
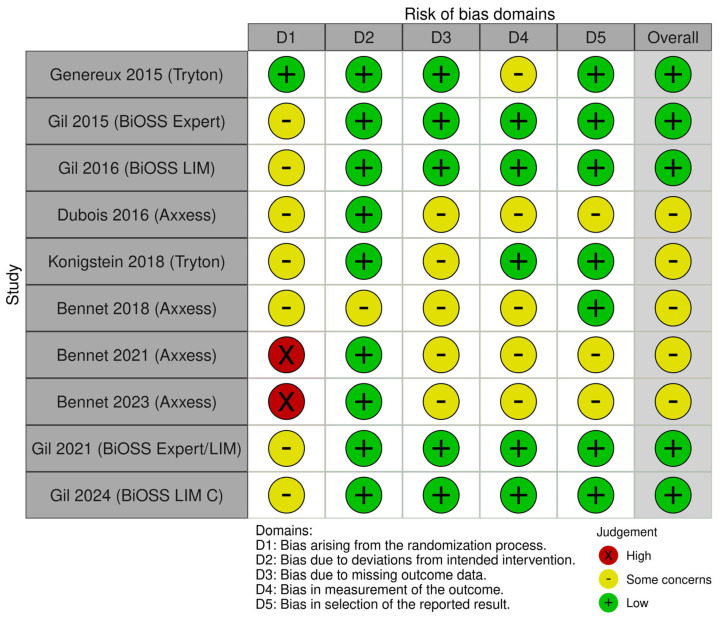
Cochrane Risk of Bias 2 (RoB 2) assessment [13,14,15,16,17,18,19,20,21,22].

The initial database search identified 653 records (PubMed: 638; Cochrane Library: 15). After removing four duplicates, 649 records underwent title and abstract screening. Of these, 633 records were excluded as irrelevant to the topic area. The full texts of 16 reports were assessed for eligibility, and six reports were subsequently excluded for not meeting the inclusion criteria. Finally, 10 studies fulfilled all eligibility requirements and were included in the quantitative meta-analysis (Figure 1). The Cochrane Risk of Bias 2 (RoB 2) assessment is shown in Figure 2.

### 3.2. Study Characteristics

A total of 10 studies involving approximately 2500 patients undergoing PCI for coronary bifurcation lesions were included, as presented in Table 2.

The POLBOS I and II trials and their pooled 6-year follow-up included 445 patients (mean age 66.4 ± 9.5 years, 28% women) with a high prevalence of hypertension (~79%), hypercholesterolemia (~70%), diabetes (28–40%), and prior myocardial infarction (MI) (~42%). Approximately 27% of lesions involved distal left main (LM) bifurcations, while the remainder were primarily LAD/diagonal bifurcations. The studies compared BiOSS Expert or BiOSS LIM dedicated stents with regular DES (sirolimus-, paclitaxel-, everolimus-, or zotarolimus-eluting) [14,15,22].

The POLBOS 3 trial enrolled 230 patients (mean age 64.3 ± 9.6 years, 19.6% women), restricted to non-LM bifurcations. Comorbidities included hypertension (79.6%), diabetes (34.8%), and prior MI (39.6%). Lesions were mainly in LAD/diagonal bifurcations (56.5%), followed by LCx (25.2%) and RCA (18.3%) [21].

The TRYTON randomized trial [13] and its pooled analysis [17] included 704 patients in the RCT and 411 in the pooled analysis, with true bifurcation lesions involving large side branches (≥2.25 mm). Mean age was 64–65 years, with 75–83% men and diabetes in ~27%. Lesions were predominantly non-LM.

The COBRA study [16] and COBRA II [20] randomized 40 and 15 patients with complex bifurcations, mostly in LAD. Patients were treated with Axxess plus Biomatrix stents versus a culotte strategy with Xience DES. Mean age was ~65 years, with 70–75% men and 20–25% diabetes prevalence. The Bennett 2023 follow-up study provided longer-term imaging and clinical outcomes for COBRA patients [19].

Collectively, these studies reflect a diverse bifurcation PCI population, ranging from simpler non-LM bifurcations (POLBOS 3) to complex LM or two-stent bifurcation strategies (COBRA), allowing for robust pooled analyses comparing DBS with regular DES.

### 3.3. Clinical Outcomes at 1 Year

Across the included trials, dedicated bifurcation stents demonstrated outcomes comparable to those of regular drug-eluting stents (rDES) at 12 months. In POLBOS I and II, the rates of major adverse cardiovascular events (MACE) were 13.3% vs. 12.2% (BiOSS Expert vs. rDES) and 11.8% vs. 15% (BiOSS LIM vs. rDES), respectively, with no significant differences. Target lesion revascularization (TLR) rates were 11.5% vs. 7.3% in POLBOS I and 9.8% vs. 9% in POLBOS II. In the TRYTON RCT, target vessel failure (TVF) occurred in 17.4% vs. 12.8% (Tryton vs. provisional rDES, *p* = 0.11). However, angiographic analysis demonstrated improved side branch patency with Tryton stents (side branch stenosis 31.6% vs. 38.6%; *p* = 0.002). The TRYTON pooled analysis confirmed non-inferiority of Tryton compared to provisional rDES for TVF (8.1% vs. 9.7%) with favorable angiographic results. In the COBRA and COBRA II studies, event rates were low, with no stent thrombosis reported and comparable clinical outcomes between Axxess and culotte strategies. Imaging endpoints suggested good stent apposition and vessel healing.

**Figure 3:**
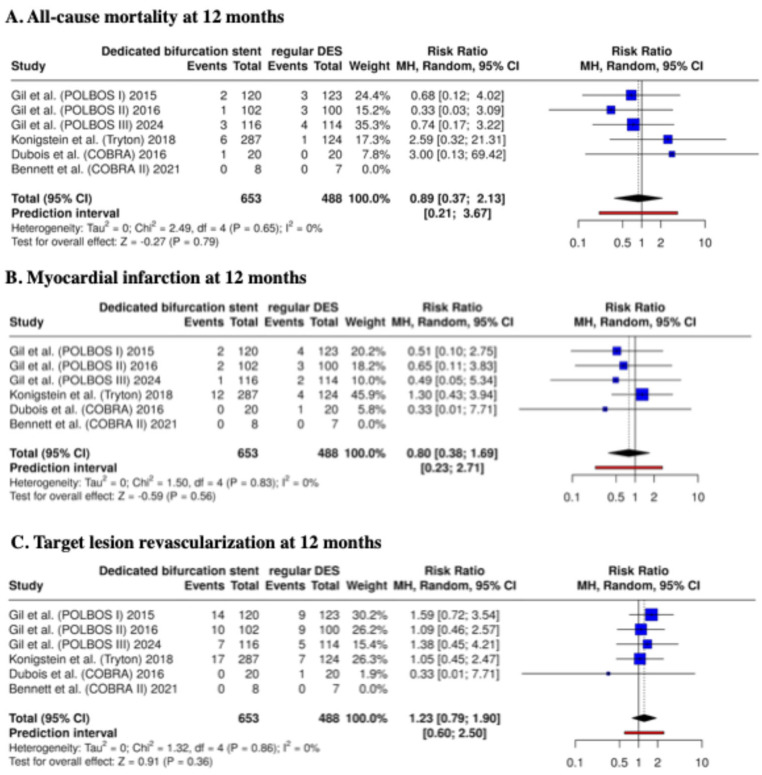
Clinical outcomes at 12 months—meta-analysis results [14,15,16,17,20,21].

The meta-analysis provided the following results. At 12 months, dedicated bifurcation stents demonstrated comparable clinical performance to regular DES. The risk of all-cause mortality did not differ significantly between the two groups (RR: 0.89; 95% CI: 0.37–2.13; *p* = 0.79; I^2^ = 0%). Similarly, the MI incidence was not significantly different (RR: 0.80; 95% CI: 0.38–1.69; *p* = 0.56; I^2^ = 0%). The TLR rate at 12 months was also comparable between dedicated bifurcation stents and regular DES (RR: 1.23; 95% CI: 0.79–1.90; *p* = 0.36; I^2^ = 0%) (Figure 3). No excess in stent thrombosis or other device-related safety concerns was observed across any study.

**Figure 4:**
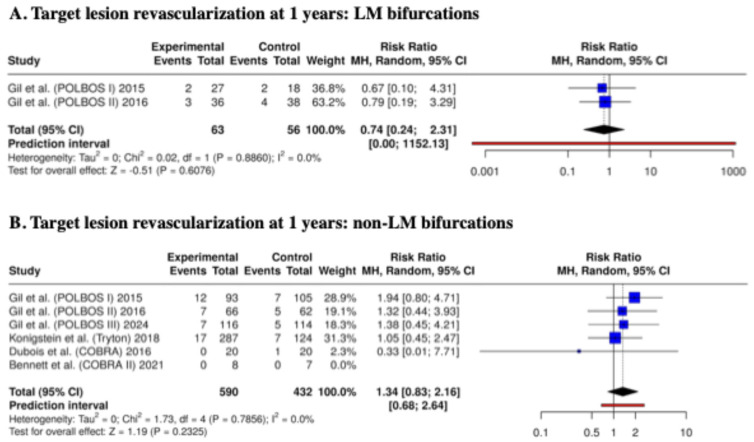
Target lesion revascularization at 12 months—meta-analysis results: LM and non-LM bifurcations [14,15,16,17,20,21].

Additionally, we analyzed TLR rates in the groups LM and non-LM (the analysis was based only on this endpoint, considering the number of events in the analyzed subgroups). In the LM bifurcation subgroup, the risk of 1-year TLR was similar between dedicated bifurcation stents and regular DES (RR: 0.74; 95% CI: 0.24–2.31; *p* = 0.61; I^2^ = 0). Also, for non-LM bifurcations, no significant difference was observed (RR: 1.34; 95% CI: 0.83–2.16; *p* = 0.23; I^2^ = 0%) (Figure 4).

### 3.4. Clinical Outcomes at 4 Years

Long-term data were primarily derived from POLBOS 3 and the pooled POLBOS I+II 6-year follow-up. In POLBOS 3 (48 months), there were no significant differences between BiOSS LIM C and rDES in terms of MACE (18.1% vs. 14.9%), cardiac death (4.3% vs. 3.5%), myocardial infarction (MI; 2.6% vs. 3.5%), or TLR (11.2% vs. 7.9%). The pooled 6-year analysis of POLBOS I and II showed comparable event rates between BiOSS and rDES: MACE (25.7% vs. 25.1%), cardiac death (3.1% vs. 4.0%), MI (3.6% vs. 4.9%), and TLR (18.9% vs. 16.1%). The COBRA 5-year follow-up [19] demonstrated sustained low MACE rates and favorable imaging findings for Axxess bifurcation stents compared with culotte stenting. No excess in stent thrombosis or late adverse events was observed across any of the dedicated bifurcation stent studies.

**Figure 5:**
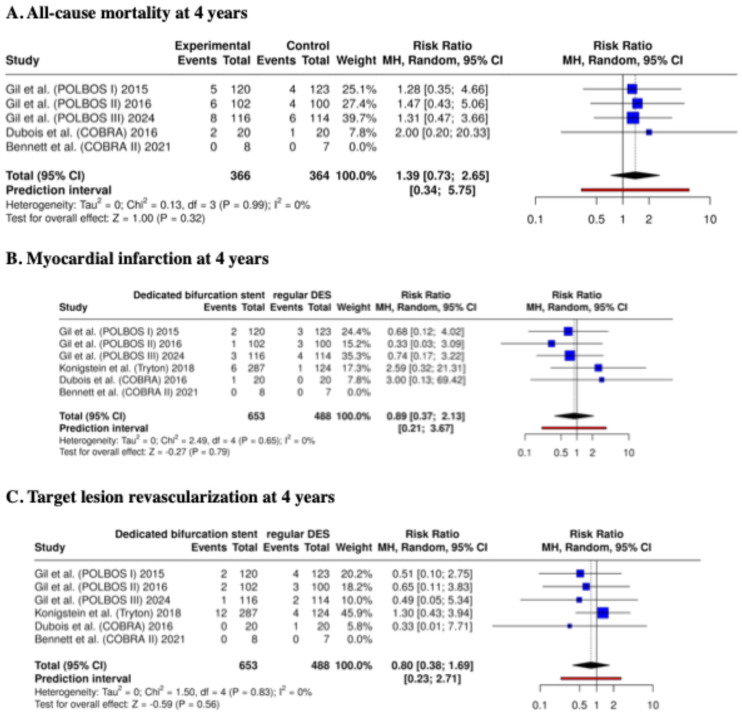
Clinical outcomes at 48 months—meta-analysis results [14,15,16,17,20,21].

The meta-analysis provided the following results. At 4 years, the results remained consistent with the 12-month findings. All-cause mortality was similar between groups (RR: 1.39; 95% CI: 0.73–2.65; *p* = 0.32; I^2^ = 0%). The risk of MI was likewise non-significant (RR: 0.66; 95% CI: 0.29–1.49; *p* = 0.32; I^2^ = 0%). For TLR, no statistically significant difference was observed between dedicated bifurcation stents and regular DES (RR: 1.29; 95% CI: 0.86–1.94; *p* = 0.22; I^2^ = 0%) (Figure 5). These findings were consistent across sensitivity and trial sequential analyses, which did not cross monitoring boundaries for statistical significance.

**Figure 6:**
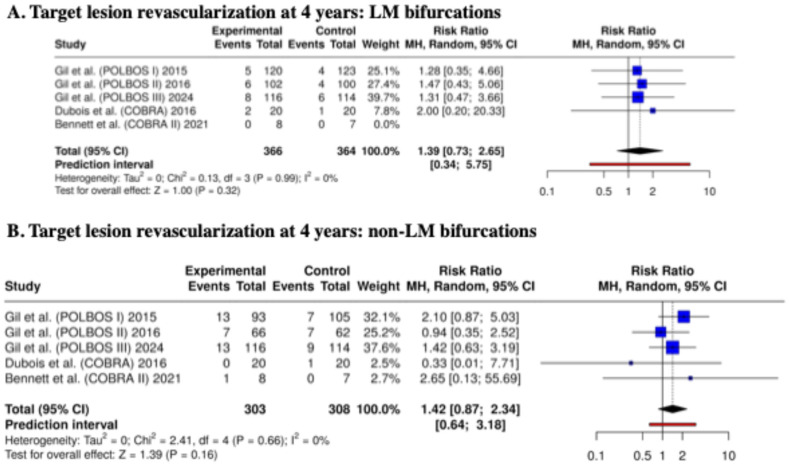
Target lesion revascularization at 48 months—meta-analysis results: LM and non-LM bifurcations [14,15,16,17,20,21].

Additionally, we analyzed TLR rates in the groups LM and non-LM (the analysis was based only on this endpoint, considering the number of events in the analyzed subgroups). In the LM bifurcation subgroup, the risk of 4-year TLR was similar between dedicated bifurcation stents and regular DES (RR: 0.85; 95% CI: 0.41–1.73; *p* = 0.65; I^2^ = 0%). Also, for non-LM bifurcations, no significant difference was observed (RR: 1.42; 95% CI: 0.87–2.34; *p* = 0.16; I^2^ = 0%) (Figure 6).

## 4. Discussion

Our meta-analysis of randomized trials demonstrates that dedicated bifurcation stents—such as BiOSS LIM C, Tryton, and Axxess—offer comparable long-term safety and efficacy to conventional drug-eluting stents (DES) in the treatment of coronary bifurcation lesions. At 12 months and 4 years, no statistically significant differences existed in all-cause mortality, myocardial infarction (MI), or target lesion revascularization (TLR), with consistency maintained across LM and non-LM bifurcation subgroups.

Percutaneous coronary intervention (PCI) in coronary bifurcation lesions remains one of the most technically demanding aspects of interventional cardiology. Bifurcation lesions account for approximately 15–20% of all PCI procedures and are associated with a significantly higher risk of procedural complications and adverse events than non-bifurcation lesions [23]. Registry data indicate that bifurcation PCI is linked to a 30–50% longer procedural time and requires 20–40% more contrast volume due to the frequent need for complex techniques such as two-stent strategies [24].

The geometric complexity of bifurcations—including acute vessel angulation, diameter mismatch between the proximal and distal main vessel, and the presence of a clinically relevant side branch (SB) in up to 70% of bifurcation lesions—often leads to suboptimal stent expansion and incomplete lesion coverage [25,26]. This, in turn, contributes to a twofold increase in restenosis risk and a 1.5-fold higher incidence of stent thrombosis compared to non-bifurcation PCI [27].

Despite major advancements in stent platforms and the use of intravascular imaging, conventional drug-eluting stents (DES) in bifurcation lesions still show target lesion revascularization (TLR) rates of approximately 5–8% at 1 year, compared with 2–4% in non-bifurcation PCI. These findings underscore the persistent challenges in optimizing outcomes for bifurcation PCI and the need for innovative device-based solutions [6,28].

Dedicated bifurcation stents (DBS) were developed to address the limitations of conventional DES in bifurcations by improving SB access, ensuring complete ostial coverage, and minimizing the need for complex two-stent techniques. Unlike standard DES, DBS platforms incorporate designs tailored to bifurcation anatomy, such as wider proximal diameters, side branch openings, and optimized radial force [5]. These features aim to reduce procedural steps, shorten procedural time, and potentially improve long-term outcomes by reducing restenosis and stent deformation.

Early studies suggested that DBS might provide superior procedural efficiency and angiographic results, with some reports showing shorter procedure times by 15–20% and reduced need for complex rewiring compared to standard DES [29]. However, subsequent randomized trials and meta-analyses have failed to demonstrate a clear clinical superiority of DBS over contemporary DES in terms of major adverse cardiovascular events (MACE) or target lesion revascularization (TLR) [5]. As a result, while DBS remain a promising technological development, their role in routine clinical practice continues to be limited pending more robust outcome data.

This evolution has been largely driven by technological advancements in conventional DES platforms, including thinner struts, enhanced flexibility, improved polymer biocompatibility, and higher resistance to strut fracture during high-pressure post-dilatation, allowing operators to safely achieve optimal expansion and apposition even in challenging bifurcation anatomy. In parallel, the systematic adoption of procedural techniques such as final kissing balloon (FKB) inflation and the proximal optimization technique (POT) or POT-side-POT technique has significantly improved both procedural safety and long-term outcomes, further narrowing the performance gap between standard DES and DBS [30,31].

The 2022 EBC consensus recommends the provisional stenting strategy with conventional DES as the default approach for most bifurcation lesions, reserving two-stent techniques for complex anatomies (e.g., Medina 1,1,1) or large SBs at risk [5]. Regarding DBS, the EBC recognizes their potential, particularly in specific anatomical scenarios, but does not recommend them as standard of care due to limited large-scale randomized trial evidence. Instead, DBS use is considered reasonable in selected cases, provided operators have expertise and familiarity with the specific device characteristics.

Our meta-analysis adds to the growing evidence base by systematically comparing dedicated bifurcation stents (DBS) with contemporary drug-eluting stents (DES) in bifurcation PCI. We observed that DBS demonstrated comparable safety and efficacy profiles to conventional DES, with no significant differences in major adverse cardiovascular events (MACE) or target lesion revascularization (TLR) during long-term follow-up.

These findings align with broader evidence regarding PCI in bifurcation lesions, where outcomes with modern DES remain challenging. Despite advances in stent platforms and procedural optimization, bifurcation PCI is still associated with higher rates of restenosis and TLR—reported in the range of 5–8% at 1 year, compared with 2–4% for non-bifurcation lesions—as well as increased rates of periprocedural myocardial infarction [32]. While DBS were designed to address these limitations through improved side branch scaffolding, simplified deployment, and better anatomical fit, the clinical evidence to date suggests that these procedural advantages have not yet translated into meaningful differences in hard outcomes compared with DES.

Nonetheless, dedicated stents may hold potential benefits in selected scenarios, such as large side-branch bifurcations or complex left main lesions with challenging anatomy, where their design could facilitate more predictable results and reduce procedural steps. Importantly, our meta-analysis provides long-term data—up to 4 years—confirming the absence of safety concerns, including late stent thrombosis, thereby supporting the durability of these devices [33].

Future research should focus on:

- Defining patient and lesion subsets most likely to benefit from DBS, particularly in bifurcations with side branches ≥ 2.25 mm or marked vessel size mismatch.
- Integrating intravascular imaging (IVUS or OCT) and procedural standardization, such as systematic POT and side branch POT, into trial designs to improve precision in bifurcation PCI.
- Evaluating newer DBS iterations with thinner struts, improved deliverability, and optimized polymer coatings (e.g., BiOSS LIM C) to determine whether these refinements can reduce restenosis and enhance vessel healing.

Although current evidence supports the safety of DBS, larger randomized trials with sufficient power are needed to detect modest but clinically meaningful differences compared to contemporary DES.

To our best knowledge, the current market offers only a limited number of DBS, each designed to address specific anatomical challenges and procedural needs in bifurcation PCI. While their core objective is to simplify treatment and improve clinical outcomes compared with conventional two-stent strategies, their clinical use has remained relatively niche, largely due to the lack of robust outcome data and the rapid evolution of contemporary DES technology.

Several DBS platforms are currently available, each with unique design characteristics:

- BIOSS LIM C (Balton): Features a stepped balloon and tapered design to match the anatomy of bifurcations better and facilitate main vessel (MV) and side branch (SB) coverage. This design allows for simplified crossover stenting and optimized proximal stent apposition. However, long-term clinical outcome data remain limited, and their adoption is still restricted mainly to specialized centers [10].
- Biomime Branch (Meril Life Sciences): Designed for a “main vessel–side branch” approach, this stent incorporates an open-cell configuration at the SB ostium to facilitate SB access while maintaining scaffold integrity in the proximal MV segment. Importantly, the Biomime Branch is used in combination with a standard DES for the main vessel, resembling the staged concept previously introduced with the Tryton stent. Although early reports on procedural feasibility are encouraging, robust long-term safety and efficacy data are still lacking [11].

Recent expert recommendations from the 2024 EBC consensus reinforce the provisional one-stent approach with contemporary DES as the default strategy for most bifurcation lesions, reserving complex two-stent techniques or dedicated bifurcation stents for anatomically demanding cases (e.g., large side branches ≥ 2.5 mm or significant vessel diameter mismatch) [4]. The consensus further emphasizes the importance of systematic proximal optimization (POT), final kissing balloon (FKB), and side-POT steps, which have markedly improved outcomes in bifurcation PCI. Within this framework, our meta-analysis supports the EBC position by demonstrating that dedicated bifurcation stents provide outcomes comparable to contemporary DES, confirming their safety while acknowledging that broad clinical superiority remains unproven.

At the same time, next-generation DBS iterations, such as the Biomime Branch stent, exemplify ongoing innovation aimed at combining optimized main-vessel scaffolding with an open-cell side-branch segment to facilitate SB access and complete ostial coverage [11]. Early clinical data indicate improved deliverability and reduced procedural time compared with earlier DBS models, though robust long-term results are still awaited. Our findings, therefore, align with the current EBC perspective that dedicated stents may offer procedural advantages in specific anatomical subsets but require further high-quality randomized evidence to define their role in everyday practice.

Worth stressing are some statistical aspects. Although statistical heterogeneity across the included trials was low (I^2^ ≈ 0%), this should not obscure the presence of important clinical and procedural heterogeneity. The analyzed studies differed in the proportion of true bifurcation lesions and in the adopted strategies—ranging from provisional single-stent techniques to pre-planned two-stent *or* complex bifurcation approaches. These variations reflect real-world diversity in bifurcation PCI and likely contributed to subtle differences in lesion selection, operator technique, and device handling that are not captured by pooled summary statistics. Because the number of RCTs directly comparing DBS and DES remains small, and reporting of procedural stratification was inconsistent, subgroup analyses according to stenting strategy or true-bifurcation status were not feasible within the current meta-analysis. Future patient-level pooled analyses are warranted to explore whether outcomes differ according to these clinically meaningful, non-statistical sources of heterogeneity.

Finally, it is important to emphasize that the lack of statistical significance in this meta-analysis should not be interpreted as evidence of clinical equivalence between dedicated bifurcation stents and regular DES. The wide confidence intervals observed across endpoints (e.g., RR for MI = 0.66, 95% CI 0.29–1.49) indicate a high degree of uncertainty driven by limited sample size, heterogeneous lesion complexity, and differing procedural strategies. These broad intervals encompass both potential benefit and potential disadvantage of DBS compared with DES, underscoring that the present findings are inconclusive rather than demonstrative of comparable performance. The observed non-significance therefore reflects insufficient power and between-study variability, reinforcing the need for adequately powered randomized trials to determine whether any true clinical differences exist.

### Study Limitations

This meta-analysis should be interpreted in light of several limitations. First, the number of available randomized controlled trials directly comparing dedicated bifurcation stents with regular DES was limited, and the overall sample size—particularly in long-term follow-up and in the left main bifurcation subgroup—remains modest. Second, there was variability in the types of dedicated bifurcation stents and control DES used across the included trials. The studies incorporated different device generations (e.g., BiOSS Expert, BiOSS LIM, Tryton, Axxess) as well as various regular DES platforms. This heterogeneity may have attenuated potential differences between individual devices. Third, intravascular imaging guidance (IVUS or OCT) was not uniformly used or reported across studies, which may have influenced procedural optimization and clinical outcomes. Fourth, this meta-analysis relied on aggregate study-level data rather than individual patient-level data, precluding adjustment for baseline imbalances or performance of detailed subgroup analyses. Finally, procedural techniques and operator experience were not consistently described, and adherence to contemporary bifurcation PCI strategies—such as proximal optimization and kissing balloon inflation—may have varied among studies.

Despite these limitations, this meta-analysis represents the most comprehensive synthesis of randomized evidence comparing dedicated bifurcation stents and regular DES to date, including both short- and long-term outcomes. It provides clinically relevant insights for the management of coronary bifurcation lesions.

## 5. Conclusions

Dedicated bifurcation stents demonstrated non-inferior safety and efficacy compared with regular DES at 12 months and 4 years. No signal of excess mortality, MI, or TLR was observed in any subgroup, including LM bifurcations. While these findings support the long-term safety of dedicated bifurcation stents, the results should be interpreted in the context of limited statistical power and wide prediction intervals, underscoring the need for larger, adequately powered randomized trials.

## Figures and Tables

**Table 1:** Search strategy for PubMed.

Search Number	Query	Sort By	Filters	Search Details
10	(coronary) AND ((((“dedicated bifurcation stent”) OR (DBS)) OR ((coronary) AND (bifurcation))) AND (randomized))	Publication Date		(“coronaries”[All Fields] OR “heart”[MeSH Terms] OR “heart”[All Fields] OR “coronary”[All Fields]) AND ((“dedicated bifurcation stent”[All Fields] OR “DBS”[All Fields] OR ((“coronaries”[All Fields] OR “heart”[MeSH Terms] OR “heart”[All Fields] OR “coronary”[All Fields]) AND (“bifurcate”[All Fields] OR “bifurcated”[All Fields] OR “bifurcates”[All Fields] OR “bifurcating”[All Fields] OR “bifurcation”[All Fields] OR “bifurcational”[All Fields] OR “bifurcations”[All Fields]))) AND (“random allocation”[MeSH Terms] OR (“random”[All Fields] AND “allocation”[All Fields]) OR “random allocation”[All Fields] OR “randomization”[All Fields] OR “randomized”[All Fields] OR “random”[All Fields] OR “randomisation”[All Fields] OR “randomisations”[All Fields] OR “randomise”[All Fields] OR “randomised”[All Fields] OR “randomising”[All Fields] OR “randomizations”[All Fields] OR “randomize”[All Fields] OR “randomizes”[All Fields] OR “randomizing”[All Fields] OR “randomness”[All Fields] OR “randoms”[All Fields]))
7	(((“dedicated bifurcation stent”) OR (DBS)) OR ((coronary) AND (bifurcation))) AND (randomized)	Publication Date		(“dedicated bifurcation stent”[All Fields] OR “DBS”[All Fields] OR ((“coronaries”[All Fields] OR “heart”[MeSH Terms] OR “heart”[All Fields] OR “coronary”[All Fields]) AND (“bifurcate”[All Fields] OR “bifurcated”[All Fields] OR “bifurcates”[All Fields] OR “bifurcating”[All Fields] OR “bifurcation”[All Fields] OR “bifurcational”[All Fields] OR “bifurcations”[All Fields]))) AND (“random allocation”[MeSH Terms] OR (“random”[All Fields] AND “allocation”[All Fields]) OR “random allocation”[All Fields] OR “randomization”[All Fields] OR “randomized”[All Fields] OR “random”[All Fields] OR “randomisation”[All Fields] OR “randomisations”[All Fields] OR “randomise”[All Fields] OR “randomised”[All Fields] OR “randomising”[All Fields] OR “randomizations”[All Fields] OR “randomize”[All Fields] OR “randomizes”[All Fields] OR “randomizing”[All Fields] OR “randomness”[All Fields] OR “randoms”[All Fields])
9	“dedicated bifurcation stent”	Publication Date		“dedicated bifurcation stent”[All Fields]
8	(((“dedicated bifurcation stent”) OR (DBS)) OR ((coronary) AND (bifurcation))) AND (randomized)	Publication Date	Randomized Controlled Trial	((“dedicated bifurcation stent”[All Fields] OR “DBS”[All Fields] OR ((“coronaries”[All Fields] OR “heart”[MeSH Terms] OR “heart”[All Fields] OR “coronary”[All Fields]) AND (“bifurcate”[All Fields] OR “bifurcated”[All Fields] OR “bifurcates”[All Fields] OR “bifurcating”[All Fields] OR “bifurcation”[All Fields] OR “bifurcational”[All Fields] OR “bifurcations”[All Fields]))) AND (“random allocation”[MeSH Terms] OR (“random”[All Fields] AND “allocation”[All Fields]) OR “random allocation”[All Fields] OR “randomization”[All Fields] OR “randomized”[All Fields] OR “random”[All Fields] OR “randomisation”[All Fields] OR “randomisations”[All Fields] OR “randomise”[All Fields] OR “randomised”[All Fields] OR “randomising”[All Fields] OR “randomizations”[All Fields] OR “randomize”[All Fields] OR “randomizes”[All Fields] OR “randomizing”[All Fields] OR “randomness”[All Fields] OR “randoms”[All Fields])) AND (randomizedcontrolledtrial[Filter])
6	((“dedicated bifurcation stent”) OR (DBS)) OR ((coronary) AND (bifurcation))	Publication Date		“dedicated bifurcation stent”[All Fields] OR “DBS”[All Fields] OR ((“coronaries”[All Fields] OR “heart”[MeSH Terms] OR “heart”[All Fields] OR “coronary”[All Fields]) AND (“bifurcate”[All Fields] OR “bifurcated”[All Fields] OR “bifurcates”[All Fields] OR “bifurcating”[All Fields] OR “bifurcation”[All Fields] OR “bifurcational”[All Fields] OR “bifurcations”[All Fields]))
5	(coronary) AND (bifurcation)	Publication Date		(“coronaries”[All Fields] OR “heart”[MeSH Terms] OR “heart”[All Fields] OR “coronary”[All Fields]) AND (“bifurcate”[All Fields] OR “bifurcated”[All Fields] OR “bifurcates”[All Fields] OR “bifurcating”[All Fields] OR “bifurcation”[All Fields] OR “bifurcational”[All Fields] OR “bifurcations”[All Fields])
4	randomized	Publication Date		“random allocation”[MeSH Terms] OR (“random”[All Fields] AND “allocation”[All Fields]) OR “random allocation”[All Fields] OR “randomization”[All Fields] OR “randomized”[All Fields] OR “random”[All Fields] OR “randomisation”[All Fields] OR “randomisations”[All Fields] OR “randomise”[All Fields] OR “randomised”[All Fields] OR “randomising”[All Fields] OR “randomizations”[All Fields] OR “randomize”[All Fields] OR “randomizes”[All Fields] OR “randomizing”[All Fields] OR “randomness”[All Fields] OR “randoms”[All Fields]
3	bifurcation	Publication Date		“bifurcate”[All Fields] OR “bifurcated”[All Fields] OR “bifurcates”[All Fields] OR “bifurcating”[All Fields] OR “bifurcation”[All Fields] OR “bifurcational”[All Fields] OR “bifurcations”[All Fields]
2	coronary	Publication Date		“coronaries”[All Fields] OR “heart”[MeSH Terms] OR “heart”[All Fields] OR “coronary”[All Fields]
1	(“dedicated bifurcation stent”) OR (DBS)	Publication Date		“dedicated bifurcation stent”[All Fields] OR “DBS”[All Fields]

**Table 2:** Procedural characteristics.

Study	N (Patients)	Age (yrs)	Female (%)	Hypertension (%)	Diabetes (%)	Prior MI (%)	LM Bifurcation (%)	Main Lesion Location	Devices Compared	Follow-up/Outcomes [DBS vs. rDES]
Gil et al., 2024 [21]	230	64.3 ± 9.6	19.6	79.6	34.8	39.6	0	LAD 56.5%, LCx 25.2%, RCA 18.3	BiOSS LIM C vs. rDES (Xience, Resolute, Orsiro, Synergy)	48 mo: MACE 18.1% vs. 14.9%; Cardiac Death 4.3% vs. 3.5%; MI 2.6% vs. 3.5%; TLR 11.2% vs. 7.9%
Gil et al., 2021 [22]	445	66.4 ± 9.5	28.3	78.9	34.4	42.5	26.7	LM 26.7%, non-LM 73.3%	BiOSS Expert/LIM vs. rDES (SES, PES, EES, ZES)	72 mo: MACE 25.7% vs. 25.1%; Cardiac Death 3.1% vs. 4.0%; MI 3.6% vs. 4.9%; TLR 18.9% vs. 16.1%
Gil et al., 2016 [15]	202	67	23.1 vs. 25	84.3 vs. 81	44.1 vs. 32	43.1 vs. 48	35.3 vs. 38	LAD 44.1/43%, LCx 15.7/15%, RCA 4.9/4%	BiOSS LIM vs. rDES	12 mo: MACE 11.8% vs. 15%; TLR 9.8% vs. 9%
Gil et al., 2015 [14]	243	66	31.2 vs. 31.7	78.3 vs. 73.2	37.5 vs. 25.2	45.8 vs. 35	22.5 vs. 15	LAD 52.5/70%, LCx 18/13%, RCA 8/2%	BiOSS Expert vs. rDES	12 mo: MACE 13.3% vs. 12.2%; TLR 11.5% vs. 7.3%
Genereux et al., 2015 [13]	704	64	28.2 vs. 26.6	73.2 vs. 73.6	23.9 vs. 28.1	30 vs. 37.8	0	LAD 75.8%; LCx 18.2%, RCA 6%	Tryton vs. provisional rDES	9 mo: TVF 17.4% vs. 12.8%; SB stenosis 31.6% vs. 38.6%
Konigstein et al., 2018 [17]	411	64.8	25.1 vs. 16.9	76 vs. 77.2	26.9 vs. 29	31.1 vs. 41	1 case in Tryton	LAD 72.5/68.5%; LCx 19.9/22.6%; RCA 7.3%/8.9%	Tryton vs. provisional rDES	12 mo: MACE 31% vs. 12%; TLR 17% vs. 7%
Dubois et al., 2016 [16]	40	65	25–30	70–75	20–25	10–30	0	LAD 97.5%; RCA 2.5%	Axxess + Biomatrix vs. culotte Xience	12 mo: MACE 10% vs. 10%; TLR 0 vs. 5%
Bennet et al., 2021 [20]	15	63	37–0	63–71	25–43	25–29	0	LAD 88/71%; LCx 12/29%	Axxess + Biomatrix vs. modified-T Absorb BVS	12 mo: MACE/TLR 12.5% vs. 0
Bennett et al., 2023 [19]	40	65	25–30	70–75	20–25	10–30	0	LAD 97.5%; RCA 2.5%	Axxess + Biomatrix vs. culotte Xience	5 yr: MACE 12% vs. 0
Bennet et al., 2018 [18]	40	65	25–30	70–75	20–25	10–30	0	LAD 97.5%; RCA 2.5%	Axxess + Biomatrix vs. culotte Xience	5 yr: MACE 0 vs. 10%; TLR 0 vs. 5%

BVS—bioresorbable vascular scaffold; DBS—dedicated bifurcation stent; DES—drug-eluting stent; EES—everolimus-eluting stent; LAD—left anterior descending coronary artery; LCx—left circumflex coronary artery; LM—left main coronary artery; MACE—major adverse cardiac events; MI—myocardial infarction; PES—paclitaxel-eluting stent; rDES—regular (conventional) drug-eluting stent; SB—side branch; SES—sirolimus-eluting stent; TLR—target lesion revascularization; TVF—target vessel failure; ZES—zotarolimus-eluting stent.

## Data Availability

Data are available from the corresponding author on request.

## Supplementary Materials

The following supporting information can be downloaded at: https://www.mdpi.com/article/10.3390/biomedicines13112763/s1: Supplementary Table S1: Study endpoints definitions per study.

## List of Abbreviations

ABPM	Ambulatory blood pressure monitoring
BMS	Bare-metal stent
BVS	Bioresorbable vascular scaffold
CAD	Coronary artery disease
CBL	Coronary bifurcation lesion
CI	Confidence interval
CTO	Chronic total occlusion
DBS	Dedicated bifurcation stent
DES	Drug-eluting stent
DK	Double kissing (technique)
EBC	European Bifurcation Club
EES	Everolimus-eluting stent
FKB	Final kissing balloon (inflation)
IVUS	Intravascular ultrasound
LAD	Left anterior descending coronary artery
LCx	Left circumflex coronary artery
LM	Left main coronary artery
MACE	Major adverse cardiac events
MI	Myocardial infarction
MV	Main vessel
OCT	Optical coherence tomography
PCI	Percutaneous coronary intervention
PES	Paclitaxel-eluting stent
POT	Proximal optimization technique
PRISMA	Preferred Reporting Items for Systematic Reviews and Meta-Analyses
RCT	Randomized controlled trial
rDES	Regular (conventional) drug-eluting stent
RR	Risk ratio
SB	Side branch
SES	Sirolimus-eluting stent
ST	Stent thrombosis
TSA	Trial sequential analysis
TLR	Target lesion revascularization
TVF	Target vessel failure
ZES	Zotarolimus-eluting stent
