# Supplementary material for: Dedicated Bifurcation Stents vs. Regular Drug-Eluting Stents in Coronary Bifurcation Treatment: A Systematic Review and Meta-Analysis of 1-Year and 4-Year Outcomes, Including Left Main and Non-Left Main Subgroup Comparisons
Source: Biomedicines. 2025 Nov 12;13(11):2763. doi: 10.3390/biomedicines13112763 (PMC12650738; doi:10.3390/biomedicines13112763)
Supplement: Supplementary file 1 [file biomedicines-13-02763-s001.zip › biomedicines-3971934-supplementary.pdf]

Supplementary Table S1: Study endpoints per definition

Study	Study endpoints
Gil et al., 2024 [21]	The primary endpoint was the rate of major cardiovascular events (cardiac death, myocardial infarction, and target lesion revascularization). The secondary endpoints were as follows: all-cause death; cardiovascular death—all deaths considered cardiovascular death if not proved otherwise; myocardial infarction; target lesion revascularization; and stent thrombosis.
Gil et al., 2021 [22]	The primary endpoint was the rate of major cardiovascular events (cardiac death, myocardial infarction, and target lesion revascularization). The secondary endpoints were as follows: all-cause death; cardiovascular death—all deaths considered cardiovascular death if not proved otherwise; myocardial infarction; target lesion revascularization; and stent thrombosis.
Gil et al., 2016 [15]	The primary endpoint was the rate of major cardiovascular events (cardiac death, myocardial infarction, and target lesion revascularization). The secondary endpoints were as follows: all-cause death; cardiovascular death—all deaths considered cardiovascular death if not proved otherwise; myocardial infarction; target lesion revascularization; and stent thrombosis.
Gil et al., 2015 [14]	The primary endpoint was the rate of major cardiovascular events (cardiac death, myocardial infarction, and target lesion revascularization). The secondary endpoints were as follows: all-cause death; cardiovascular death—all deaths considered cardiovascular death if not proved otherwise; myocardial infarction; target lesion revascularization; and stent thrombosis.
Genereux et al., 2015 [13]	The primary endpoint (powered for noninferiority) at 9-month follow-up was the rate of target vessel failure (TVF) defined as the composite of cardiac death, target vessel MI (Q-wave or non–Q-wave [3 upper limit of normal creatine kinase {CK}-MB]), and clinically driven target vessel revascularization (TVR) in the MB or SB. The secondary angiographic endpoint (powered for superiority) was the SB in-segment percent diameter stenosis of the bifurcation stent compared with SB balloon angioplasty at the 9-month follow-up. Pre-specified additional clinical secondary endpoints included the following: the rates of device success (<30% residual stenosis within the SB), lesion success (<50% residual stenosis using any percutaneous method), and procedural success (lesion success without the occurrence of in-hospital major adverse cardiac events [death, MI, emergent coronary artery bypass graft, clinically driven target lesion revascularization]); the rate of all-cause and cardiac mortality; the rate of Academic Research Consortium–defined stent thrombosis; and the rate of target lesion revascularization.
Konigstein et al., 2018 [17]	The primary endpoint of this study was the rate of target vessel failure (TVF), defined as the composite of cardiac death, target vessel MI (The Society for Cardiovascular Angiography and Interventions [SCAI] definition), and clinically driven target vessel revascularization in the MB or SB at 1 year. Pre-specified additional clinical secondary endpoints included the following: The rates of device success (<30% residual stenosis within the SB), lesion success (<50% residual stenosis using any percutaneous method), and procedural success (lesion success without the occurrence of in-hospital major adverse cardiac events [MACEs; death, MI, emergent coronary artery bypass grafting, clinically driven target lesion revascularization]); the rate of all-cause and cardiac mortality; the rate of Academic Research Consortium defined stent thrombosis, and the rate of target lesion revascularization.
Dubois et al., 2016 [16]	The clinical endpoints of the study were reported for descriptive purposes only and included the rate of major adverse cardiac events (MACE) and their components, all-cause death, target vessel revascularisation (TVR), non-target vessel revascularisation (non-TVR), and stent thrombosis at one month, eight months and one year. MACE were defined as any of the following: cardiac death, myocardial infarction (MI) and ischaemia-driven target lesion revascularisation (TLR).
Bennet et al., 2021 [20]	The clinical endpoints of the study are reported for descriptive purposes only and include the major adverse cardiac event (MACE) rate and the components of all-cause death, target-vessel revascularization (TVR), non-target vessel revascularization (non-TVR), and stent thrombosis. MACE is defined as any of the following: cardiac death, myocardial infarction (MI), and ischemia-driven target-lesion revascularization (TLR). All deaths are considered cardiac unless an unequivocal noncardiac cause can be demonstrated. MI is defined as evidence of myocardial necrosis in a clinical setting consistent with myocardial ischemia. This will require detection of

	rise of cardiac biomarkers with at least one value above the 99th percentile of the upper reference limit (URL) together with symptoms of ischemia, or pathognomonic electrocardiographic or imaging evidence of ischemia. By convention, increases of cardiac biomarkers above 3x the URL will be used to define PCI-related MI. Repeat revascularization for restenosis of the index bifurcation lesion (TLR) will only be performed if there is significant angiographic restenosis (70% diameter stenosis anywhere within the target lesion) in combination with clear angina or a fractional flow reserve (FFR) <0.80 in the MV or a SB subtending a large myocardial territory. Stent thrombosis is defined according to the Academic Research Consortium criteria.
Bennett et al., 2023 [19]	The clinical endpoints of the study are reported for descriptive purposes only and include the major adverse cardiac event (MACE) rate and the components of all-cause death, target-vessel revascularization (TVR), non-target vessel revascularization (non-TVR), and stent thrombosis. MACE is defined as any of the following: cardiac death, myocardial infarction (MI), and ischemia-driven target-lesion revascularization (TLR). All deaths are considered cardiac unless an unequivocal noncardiac cause can be demonstrated. MI is defined as evidence of myocardial necrosis in a clinical setting consistent with myocardial ischemia. This will require detection of rise of cardiac biomarkers with at least one value above the 99th percentile of the upper reference limit (URL) together with symptoms of ischemia, or pathognomonic electrocardiographic or imaging evidence of ischemia. By convention, increases of cardiac biomarkers above 3x the URL will be used to define PCI-related MI. Repeat revascularization for restenosis of the index bifurcation lesion (TLR) will only be performed if there is significant angiographic restenosis (70% diameter stenosis anywhere within the target lesion) in combination with clear angina or a fractional flow reserve (FFR) <0.80 in the MV or a SB subtending a large myocardial territory. Stent thrombosis is defined according to the Academic Research Consortium criteria.
Bennet et al., 2018 [18]	The clinical end-points include the rate of major adverse cardiac events (MACE) and their components [cardiac death, myocardial infarction (MI) and ischemia-driven target lesion revascularization (TLR)], all-cause death, target vessel revascularization (TVR), non-target vessel revascularization (non-TVR), non-TLR, and stent thrombosis during follow-up. Peri-procedural MI was arbitrarily defined by the elevation of cardiac enzymes (>5 URL for troponin; >3 URL for CKMB) and in addition clinical evidence of MI (Universal Definition).
